# INTERACTIONS BETWEEN SLEEP AND BIOLOGICAL MARKERS OF AGING

**DOI:** 10.1093/geroni/igac059.1435

**Published:** 2022-12-20

**Authors:** Christopher Kaufmann, Katie Stone

## Abstract

There is well documented evidence that sleep architecture and circadian patterns change as people age in part due to accumulation of comorbidities, polypharmacy, and other factors inherent to the aging process. While a number of studies have examined sleep in the context of these factors, recent advances in the assessment of biological aging (including epigenetic age, metabolomics, and measurement of inflammatory biomarkers) have made it possible to identify potential mechanisms by which sleep impacts the aging course. In this symposium, we will highlight research that investigates the link between sleep disturbances and biological factors in order to identify whether sleep could be a modifiable risk factor for accelerated aging. Our first presentation will examine the association between insomnia symptoms and short sleep duration with epigenetic age acceleration using data from the Health and Retirement Study. Second, we will describe whether objectively measured sleep characteristics are associated with a number of metabolomic markers of aging. The third presentation will center around the extent to which social disparities in presence of inflammatory biomarkers may be mediated by sleep quality. Finally, we will examine how daytime sleepiness may be associated with longitudinal BMI trajectories. These presentations will, as a whole, highlight the importance of sleep as a contributor to healthy aging and longevity, and inform the development of interventional approaches targeting biological mechanisms to promote successful aging more broadly.

